# The design and psychometric evaluation of a COVID-19 social stigma questionnaire in nurses

**DOI:** 10.1186/s12912-023-01620-2

**Published:** 2023-12-06

**Authors:** Narges Rahmani, FatemehSadat SeyedNematollah Roshan, Majedeh Nabavian, Hossein Alipour

**Affiliations:** 1 Department of Nursing , Comprehensive Health Research Center, Babol Branch, Islamic Azad University, Babol, Iran; 2 Department of Nursing, Tehran Medical Sciences Branch, Islamic Azad University, Tehran, Iran; 3 Disaster Management and Medical Emergencies Center, Mazandaran University of Medical Sciences, Sari, Iran

**Keywords:** Instrumentation, Validation, Stigma, Nurse, COVID-19

## Abstract

**Background:**

The patient’s fear of social reactions, the disease stigma, and being a transmission agent is a psychological and social consequence of contracting some diseases, especially infectious ones, in any society. The present study aimed to design and psychometrically evaluate a COVID-19 social stigma questionnaire in nurses.

**Methods:**

This mixed-method study was conducted using a sequential exploratory approach according to the Creswell method in Mazandaran Province (Iran) during 2021-22. The study was performed in three phases: (1) a qualitative phase to explain the key concept, (2) designing the scale items, and (3) an experimental phase with the scale psychometric evaluation. In the first phase, nurses’ experiences regarding the concept of COVID-19 social stigma were evaluated using a qualitative method with an inductive qualitative content analysis approach. In this phase, the lived experiences of 12 nurses working at hospitals of Babol University of Medical Sciences were extracted through in-depth interviews with semi-structured questions and analyzed by conventional content analysis. The main classes are contradictory feelings, rejection, and adaptation strategies. In the second phase, the designed items were validated by determining face validity, content validity, and construct validity using exploratory factor analysis (EFA). In addition, the scale’s reliability was determined through internal consistency and stability.

**Results:**

Following the study’s first phase, a pool of questions with 64 initial items was formed. After evaluating face and content validity, the number of items was reduced to 24 cases. An excellent total content validity (S-CVI/Ave) of 0.93 was calculated for the scale. According to EFA outputs, three factors accounted for the most variance (52.82%), and four items were excluded in this phase. The Kaiser-Meyer-Olkin (KMO) statistic and Bartlett’s test of sphericity were calculated at 0.776 and P < 0.001, respectively. The results of Cronbach’s alpha (0.796) and intraclass correlation (0.793) indicated the correlation and internal consistency of the scale.

**Conclusion:**

This scale can help healthcare managers and policymakers apply necessary protective measures by evaluating the social stigma of COVID-19 in nurses and emerging infectious diseases that may occur in the future.

**Supplementary Information:**

The online version contains supplementary material available at 10.1186/s12912-023-01620-2.

## Background

The coronavirus is a severe acute respiratory syndrome that has newly emerged as a zoonotic agent between humans and animals. The virus appeared in December 2019 and caused COVID-19 [1, 2]. Since then, the high spread rate of the virus has evoked many concerns in most countries of the world [3]. The World Health Organization (WHO) declared the disease outbreak an international health crisis in January 2020. After SARS and MERS viruses, the new coronavirus is the third pandemic caused by coronaviruses, leading to global panic [4].

According to official reports, 6,398,412 people worldwide died from this epidemic until August 2022 [5]. The virus has infected Iran the same as other countries, and the fight against this virus is ongoing throughout the country [6]. The first case of COVID-19 in Iran was observed in Qom on February 18, 2020, after which the prevalence and death rate of the disease increased dramatically [7].

The patient’s fear of social reactions, the disease stigma, and being a transmission agent are among the psychological and social consequences of contracting some diseases, especially infectious ones. This fear is an important issue that has not received due attention, considering the heavy attack of the disease on different aspects of society. The current COVID-19 spread has caused stigma and socially discriminatory behaviors against carriers and infected people. Moreover, social stigma can cause behavioral and psychological disorders and negatively influence patients, doctors, nurses, and the patient’s family [8].

Stigma refers to a trait that discredits a person in others’ eyes and changes the mentality from a perfect and normal person to an insignificant person as a burden on society [9]. Stigma is related to a combination of three problems: low knowledge (ignorance and misinformation), negative attitudes (prejudice), and exclusion or avoidance behaviors (discrimination) [10]. According to experts, people’s misconceptions, using words with negative connotations, disseminating false information, and misinterpreting are among the significant causes of social stigma [11]. In the health context, social stigma is the negative connection of people with a person or a group of people who share specific characteristics of a certain disease or suffer from the disease [12].

Research across different countries has shown that the first suspects with confirmed COVID-19 experienced a huge level of anxiety [13]. People’s fear of being suspected and possible cases of COVID-19 led to a negative stigma against them. This stigma was formed as rejection and discrimination from society. Health workers (e.g., nurses), as part of the core pillars of COVID-19 disaster management in health services, are also exposed to this stigma. The stigma experienced by health workers resulted in unacceptance by the community around the neighborhood or rented boarding houses [14]. Research conducted in India and Singapore indicates that social stigma is among health workers’ mental and emotional distress in treating COVID-19 patients [15]. According to the common experiences of people, this stigma is more observed toward colleagues or friends [14]. Social stigma causes symptoms such as fear, anxiety, and depression, both in ordinary people and in medical and therapeutic staff. This stigma leads to mental and psychological abnormalities, weakening of the immune system, and reduced body’s ability to fight diseases in society, including nurses as the main element of the treatment team [8, 16]. In such incidents, healthcare workers encounter social isolation, public harassment, and expulsion from public transportation and place of residence. These events can negatively affect healthcare workers’ mental health and caregiving quality [17].

Nurses’ psychophysical health is directly related to the quality of care they give to patients, improved satisfaction, work efficiency, and interest in work. Therefore, it is essential to discover nurses’ experiences and design suitable tools to support their mental health according to health needs [14, 18].

Reducing the social stigma of COVID-19 is necessary to maintain nurses’ mental health and minimize adverse consequences in this group [19]. In this respect, the accurate assessment of social stigma in nurses of COVID-19 ICUs requires a valid and reliable tool. Such a tool can help accurately assess nurses’ social stigma and improve their mental health and performance. Previous studies focused on the design of social stigma tools for the general population or healthcare workers [20, 21]. However, no COVID-19 social stigma scale has been designed for nurses of COVID ICUs, who are in direct contact with patients. The studies conducted in this context only reported some stigma characteristics, such as discrimination of family members [22], and did not comprehensively investigate social stigma characteristics in nurses. Therefore, the accurate assessment of COVID-19 social stigma in nurses requires the design of a specific questionnaire consisting of different dimensions to provide a suitable view of COVID-19 social stigma in nurses. Hence, a qualitative study is required to comprehensively examine nurses’ experiences in COVID ICUs about social stigma to further detail the specific aspects of social stigma in nurses. In this context, the WHO declares that the tools made in other countries should not be merely used with a direct and unsupervised translation; instead, it recommends developing the tools according to the language and culture of that country [23]. The social stigma during the pandemic can further spread the virus and make it difficult to control.

On the other hand, nurses also experienced social stigma [24]. This study examines whether the stigma assessment tools, with some modification, can be used to measure the social stigma in nurses during non-COVID-19 pandemics. Considering the lack of such tools in Iran, the present study aimed to design and psychometrically evaluate a COVID-19 social stigma questionnaire in nurses.

## Methods

This mixed method study was performed with a sequential exploratory approach according to the Creswell method in Mazandaran Province (Iran) during 2021-22. This method has three phases: (1) a qualitative phase to explain the key concept, (2) designing the instrument items, and (3) an experimental phase with instrument psychometrics. In the first phase, nurses’ experiences regarding the concept of COVID-19 social stigma were evaluated using a qualitative method with a phenomenological approach. According to the research objective, the participants were selected based on purposive sampling. The selection process of the samples continued until no new data appeared in the data collection process. In other words, the data reached saturation with the entry of 12 nurses. Data were collected using unstructured and in-depth interviews during which nurses expressed their experiences regarding this phenomenon. The texts of interviews were written down and analyzed simultaneously and continuously with the conventional content analysis recommended by Graneheim and Lundman. The interview used in the study has previously been published elsewhere [25]. The main classes are (1) contradictory feelings (mental tension and positive attitude), (2) rejection (being isolated and rejected in all aspects), and (3) adaptation strategies (self-awareness over time and the effective role of media).

In the second phase, a pool of questions was formed from the thematic phrases extracted from the unstructured interviews with nurses in the first phase with 64 preliminary items. The tool reflects various aspects of the nurses’ opinions in caring for COVID-19 patients in the context of their culture and value system. Then, the items extracted in two sessions were examined by the research team, and the items of the initial questionnaire were reduced to 41 items by merging the items with overlapping concepts. The items were arranged in three constructs (subscales) of rejection (15 items), social well-being (17 items), and psychological tensions (9 items) on a 4-point Likert scale (“completely disagree”, “disagree”, “agree”, “completely agree”).

In the third phase, the questionnaire was validated with three methods, namely face validity, content validity, and construct validity. Face validity (qualitative and quantitative) was measured first because the possible need for changing the sentences and phrases might change the whole validity of the questionnaire [26]. The face validity was determined qualitatively through face-to-face interviews with 10 nurses experienced in COVID-19 wards. They were asked to comment on the grammar, spelling of words, the clear concepts of items, and the ease of tool completion. After the face validity assessment of the questionnaire, two items (12 and 21) were removed from the total of 41 items due to gaining an impact score of < 1.5. As a result, the items were reduced to 39 items according to the research team’s decision.

Quantitative face validity was evaluated using the quantitative item impact method. For this purpose, 10 nurses working at COVID-19 wards were asked to express their opinions on the importance of each item using a 5-point Likert scale, namely “completely important (= 5 points), somewhat important (= 4), moderately important (= 3), slightly important (= 2), and not important at all (= 1). The average importance scores of the items with an impact score of > 1.5 were considered appropriate [26]. In this stage, items with an impact score of < 1.5 were removed after reviews with the research team’s opinion.

Content validity was determined using both qualitative and quantitative methods. Polit (2016) states that content validity should be evaluated by the judgment of experts with knowledge of instrumentation [26]. Thus, the qualitative content validity was evaluated using the opinions of 10 faculty members with rich knowledge and experience in instrumentation and research in COVID-19. They were asked to comment on each item in terms of grammar, wording, item allocation, and scaling. After qualitative content validation by experts and merging some items, 31 items were included in the quantitative content validity phase.

Content validity was evaluated quantitatively using two criteria, namely the content validity ratio (CVR) and content validity index (CVI). To determine the CVR, 10 experts with research experience on COVID-19, nursing, stigma, and work experience in the field of instrumentation were asked to examine each item based on a 3-point Likert scale (“necessary”, “useful but not necessary”, and “not necessary”). Then, the obtained values were calculated according to the formula and compared with the Lawshe standard. Finally, the minimum CVR value of 0.62 was determined for 10 people [27].

The CVI was assessed using the Waltz and Bausell criteria by calculating the item CVI (I-CVI) for each item and estimating the scale CVI (S-CVI) for the entire tool [28]. The designed questionnaire was then provided to 10 experts to determine the relevance of each item in the questionnaire on a 4-point Likert scale for each item. Afterward, the score for each item was calculated by dividing the number of experts who agreed with the items rated 3 and 4 by the total number of experts. Finally, the items with a score of < 0.7 were considered unacceptable and excluded from the tool [26]. The CVR determination provided a minimum value of 0.62 for 10 experts, excluding five items with scores lower than the standard.

In the CVI determination, two items were scored much lower than 0.7, which were considered unacceptable according to the criteria and removed after examination by the research team. An S-CVI/Ave value of 0.93 was calculated for the whole scale. After removing seven items from the total of 31 items in the content validity phase, 24 items remained for the next stage, i.e., determining the construct validity and the final reliability.

The construct validity was determined with the exploratory factor analysis (EFA). In this process, the Kaiser-Meyer-Olkin (KMO) Measure of Sampling Adequacy test was performed to determine sampling adequacy (> 0.7 is appropriate), Cruet-Bartlett’s sphericity test to identify the significance of the correlation matrix obtained with the zero hypothesis, Principle-axis Factoring (PAF) and Scree Plot to determine the number of factors comprising the questionnaire [26] on 24 items. A turning point of 0.4 was considered as the minimum factor loading required to maintain the item in the extracted factors, with an eigenvalue value of > 1. Therefore, the required number of samples is 5–10 times the tool items for factor analysis. These items are included randomly in the study from the participants of the research environment [26]. In this study, a sample of nurses with work experience at COVID wards was randomly selected with 5.5 times the number of items, including the probability of 10% attrition, i.e., 130 people. The items were randomly completed by the samples, with a response rate of 100%.

In the final phase, the reliability of the questionnaire was determined by measuring Cronbach’s alpha coefficient and the Intraclass Correlation Coefficient (ICC). For this purpose, 30 nurses were asked to complete a 20-item questionnaire. Cronbach’s alpha represents the fit level of a group of items measuring a construct, and a value between 0.7 and 0.8 represents good and sufficient internal consistency [26]. Quantitative data were analyzed using descriptive and analytical statistics with SPSS software version 22.

## Results

**Fig. 1:**
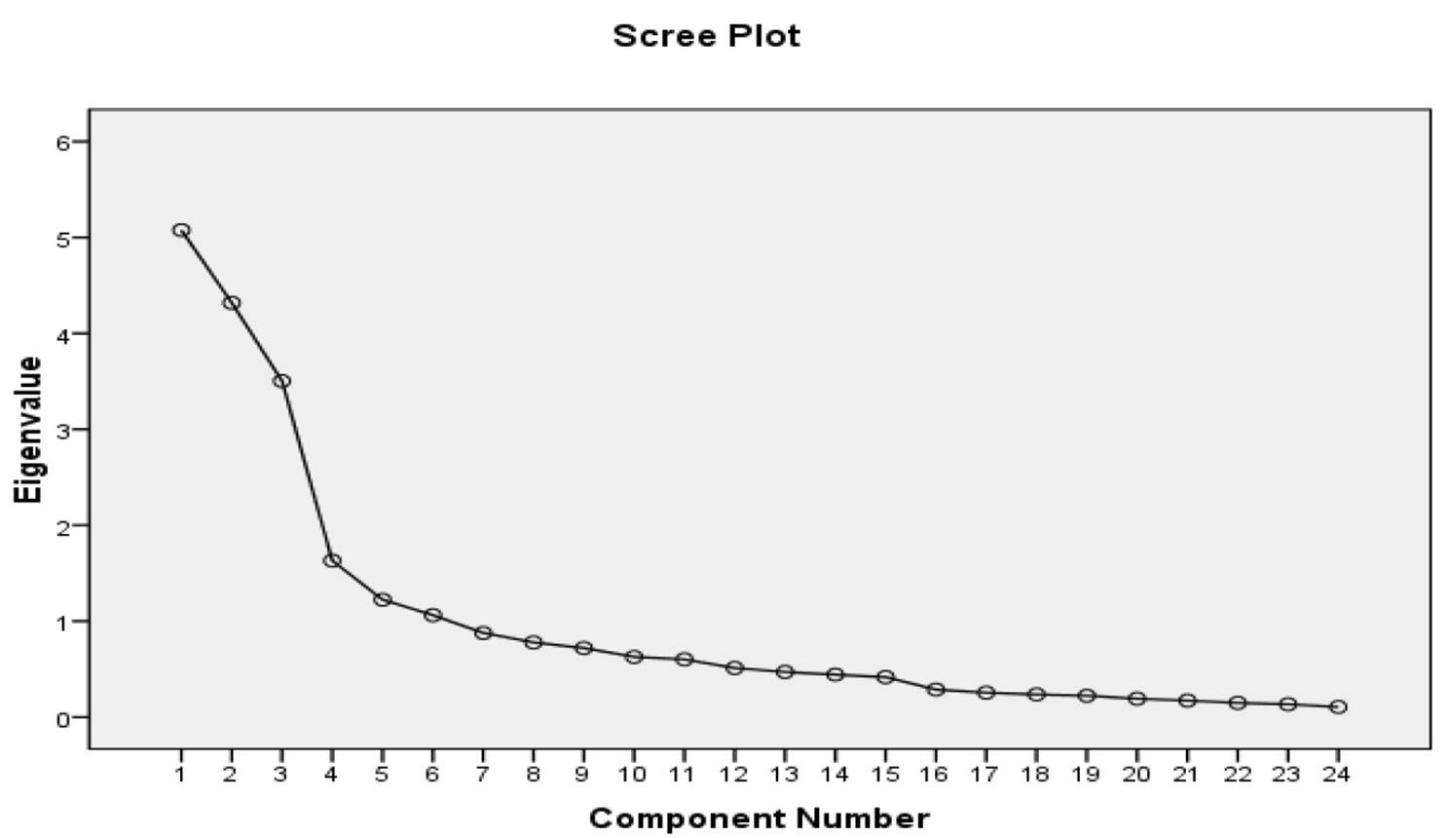
Factor analysis: Scree plot to determine the number of factors comprising the scale

The EFA method was performed on 24 statements, and a KMO value of 0.776 was obtained using the principal component analysis (PCA). Bartlett’s sphericity test was significant (1.790) at the 0.0001 level. This output justifies the EFA implementation based on the correlation matrix obtained in the studied sample (Table 1). In this research, the factors were extracted using the PCA method, and the number of factors was determined using the eigenvalue method. The results showed the presence of 17 factors with an eigenvalue of > 1. In this respect, three factors accounted for more variance than the others, with 52.82% of the explained changes belonging to the first three factors. In other words, 18.78%, 18.47%, and 15.56% of the common variance are explained by the first, second, and third factors, respectively (Table 2). The scree plot also confirms the choice of these three factors. From the third factor onward, the factors are almost at the same level with very close eigenvalues (Fig. 1).

**Table 1:** Factor analysis (KMO’s measure of sampling and the results of Bartlett’s test of sphericity)

Kaiser-Meyer-Olkin Measure of Sampling Adequacy.		0.776
Bartlett’s Test of Sphericity	Approx. Chi-Square	1.790E3
	Df	276
	Sig.	0.000

**Table 2:** Factor analysis (the total variance explained)

Component	Initial Eigenvalues			Extraction Sums of Squared Loadings			Rotation Sums of Squared Loadings		
	Total	% of variance	Cumulative %	Total	% of variance	Cumulative %	Total	% of variance	Cumulative %
1	5.076	21.152	21.152	5.076	21.152	21.152	4.508	18.785	18.785
2	4.319	17.994	39.146	4.319	17.994	39.146	4.434	18.476	37.262
3	3.503	14.594	53.740	3.503	14.594	53.740	3.734	15.559	52.820
4	1.631	6.794	60.534	1.631	6.794	60.534	1.529	6.372	59.192
5	1.223	5.098	65.632	1.223	5.098	65.632	1.308	5.450	64.642
6	1.062	4.424	70.056	1.062	4.424	70.056	1.299	5.414	70.056
7	0.877	3.653	73.709						
8	0.778	3.240	76.949						
9	0.719	2.996	79.945						
10	0.627	2.612	82.557						
11	0.601	2.503	85.060						
12	0.512	2.131	87.192						
13	0.470	1.959	89.150						
14	0.441	1.840	90.990						
15	0.416	1.735	92.725						
16	0.286	1.194	93.918						
17	0.254	1.058	94.977						
18	0.235	0.980	95.957						
19	0.222	0.926	96.883						
20	0.191	0.796	97.679						
21	0.171	0.714	98.393						
22	0.148	0.616	99.009						
23	0.133	0.554	99.564						
24	0.105	0.436	100.000						

Extraction Method: Principal Component Analysis (PCA)

A minimum acceptable factor loading of 0.4 was specified in this research, and it was tried to maintain all the items in all three factors. However, four items (i.e., 9, 10, 14, and 24) were included in none of the factors. All these four items were removed because there were alternative and overlapping items for them, and their absence led to the removal of no classes. After extracting the factors, each was named based on the variables (items). Also, their degree of compatibility with the concepts and dimensions of the COVID-19 social stigma scale was evaluated in nurses. The first factor, with seven items (a minimum acceptable factor loading of 0.686), was entitled “social well-being”. It was considered appropriate because it was more related to the nurse’s positive mentality of others’ behaviors (Table 3). The title “rejection” was considered to be appropriate for the second factor consisting of seven items (a minimum acceptable factor loading of 0.625). The explanation is that it was mostly related to the concern and stress of the nurses working at COVID wards. They felt avoidance from their friends and relatives, i.e., rejection because of more exposure to COVID-19 patients (Table 4). The third factor, i.e., “psychological tensions”, consists of six items (a minimum acceptable factor load of 0.633) since these items focused on one’s fear and discomfort concerning working at COVID-19 wards. It is of note that item 8 was initially placed in the rejection class but placed in the third factor after the factor analysis. This displacement was accepted based on the authors’ agreement regarding the convergence of this concept with the other concepts in this factor (Table 5). Finally, the reliability of the 20-item instrument was determined using internal consistency and stability methods. Thus, 30 nurses with work experience at COVID-19 wards were randomly asked to complete a 20-item questionnaire [26]. A suitable Cronbach’s alpha (> 0.7) was calculated for each dimension and the whole scale (Table 6). The reliability and stability were determined using the intra-cluster correlation index. This index was more than 0.7 for each of the dimensions and the whole scale (P < 0.01), indicating the appropriate reliability of the COVID-19 social stigma assessment scale in nurses (Table 7).

**Table 3:** Factors extracted from factor analysis using Varimax rotation (The first factor: social well-being)

Question No.	Item	Factor load
16	I am valued by the people around me.	0.901
13	The increased knowledge level of people around me made them treat me well.	0.823
15	Media propaganda has led to people’s positive attitudes toward me.	0.800
11	Patients are grateful to us as nurses in the COVID-19 ward.	0.785
17	The good treatment of colleagues motivates me to work.	0.765
12	I can talk to close people about my job because they understand me.	0.689
18	Working in the COVID-19 ward made me known as a brave person in the eyes of those around me.	0.686

**Table 4:** Factors extracted from factor analysis using Varimax rotation (The second factor: Rejection)

Question No.	Item	Factor load
4	My family stays away from me due to fear of contracting COVID-19.	0.872
3	My friends stigmatize me as being infected with COVID-19.	0.862
2	My friends stay away from me because of working in the COVID-19 ward.	0.856
5	I prefer that others do not know about my work in the COVID-19 ward.	0.820
1	Colleagues from other wards ran away from me for fear of the disease transmission.	0.774
7	I prefer to spend my free time with my colleagues because we are exposed to the same kind of infection.	0.672
6	I have decided to quit because I am forced to work in the COVID-19 ward.	0.625

**Table 5:** Factors extracted from factor analysis using Varimax rotation (The third factor: Psychological tensions)

Question No.	Item	Factor load
23	Working in the COVID-19 ward has made me angry.	0.899
19	The inappropriate behavior of people around me makes me angry and upset.	0.848
8	My children stay away from me because of the fear of contracting COVID-19.	0.772
21	I am embarrassed to work in the COVID-19 ward.	0.740
22	I am afraid of infecting the people around me with the COVID-19 virus.	0.732
20	Working in the COVID-19 department has made me feel lonely and isolated.	0.633

**Table 6:** Cronbach’s alpha coefficient of the scale for 20 items (number of samples = 30 nurses)

Dimensions	Scale Mean if Item Deleted	Scale Variance if Item Deleted	Corrected Item-Total Correlation	Cronbach’s Alpha if Item Deleted
social well-being (7 items)	93.1667	79.316	0.832	0.774
Rejection (7 items)	92.8000	84.097	0.708	0.813
Psychological tensions (6 items)	95.7000	89.114	0.787	0.818
The whole scale	56.3333	29.816	1.000	0.796

**Table 7:** The intraclass correlation coefficient of each dimension and the whole scale

		Intraclass Correlation^a^	95% Confidence Interval		F Test with True Value 0			
Dimensions	Intraclass Correlation Coefficient		Lower Bound	Upper Bound	Value	df1	df2	Sig
social well-being (7 items)	Single Measures	.644^b^	0.374	0.813	4.614	29	29	0.000
	Average Measures	.783^c^	0.545	0.897	4.614	29	29	0.000
Rejection (7 items)	Single Measures	.576b	0.278	0.773	3.719	29	29	0.000
	Average Measures	.731c	0.435	0.872	3.719	29	29	0.000
Psychological tensions (6 items)	Single Measures	.505b	0.183	0.729	3.041	29	29	0.002
	Average Measures	.671c	0.309	0.843	3.041	29	29	0.002
The whole scale	Single Measures	.143b	0.081	0.254	4.838	29	638	0.000
	Average Measures	.793c	0.669	0.887	4.838	29	638	0.000

Two-way mixed effects model where people’s effects are random, and measures effects are fixed

a. Type C intraclass correlation coefficients using a consistency definition; the between-measure variance is excluded from the denominator variance.

b. The estimator is the same, whether the interaction effect is present.

c. This estimate is computed assuming the interaction effect is absent because it is not estimable otherwise.

### Score calculation of the COVID-19 social stigma scale in nurses

In the questionnaire proposed in this study, the measurement scale was determined as a 4-point Likert ranging from negative to positive according to the accuracy and simplicity of answering [26]. Based on the experts’ opinions, when determining the content validity of the scale, the items in the dimension of social well-being were scored positively and directly as “completely agree” [4], “agree” [3], “disagree” [2], and “completely disagree” [1]. The items in two dimensions of rejection and psychological tensions were scored negatively and inversely as “completely agree” [1], “agree” [2], “disagree” [3], and “completely disagree” [4]. The scoring range was set at three levels, namely “unfavorable”, “moderate”, and “favorable”. The maximum and minimum scores belong to favorable and unfavorable choices, respectively. The social stigma of COVID-19 level in nurses generally varies between 20 and 80% of the score. The score of each subscale of social well-being, rejection, and psychological tensions is determined by calculating the average scores of the subscale items. Finally, the scale’s total score is determined by calculating the average total scores of all items. Here, the scores between 20 and 39.9, 40 and 60, and 60 and 1.80 are classified in the unfavorable, medium, and favorable ranges, respectively. Table 8 shows the social stigma scale of COVID-19 in nurses and Table 9 shows the range of scores for each class of the scale.

**Table 8:** Social stigma scale of covid-19 in nurses

Dimensions	Question No.	Item	completely disagree (1)	disagree (2)	agree (3)	completely agree (4)
social well-being	1	I am valued by the people around me				
	2	The increased knowledge level of people around me made them treat me well				
	3	Media propaganda has led to people’s positive attitudes toward me.				
	4	Patients are grateful to us as nurses in the COVID-19 ward.				
	5	The good treatment of colleagues motivates me to work.				
	6	I can talk to close people about my job because they understand me.				
	7	Working in the COVID-19 ward made me known as a brave person in the eyes of those around me.				
Rejection	8	My family stays away from me due to fear of contracting COVID-19	**(4)**	**(3)**	**(2)**	**(1)**
	9	My friends stigmatize me as being infected with COVID-19.				
	10	My friends stay away from me because of working in the COVID-19 ward.				
	11	I prefer that others do not know about my work in the COVID-19 ward.				
	12	Colleagues from other wards ran away from me for fear of the disease transmission				
	13	I prefer to spend my free time with my colleagues because we are exposed to the same kind of infection.				
	14	I have decided to quit because I am forced to work in the COVID-19 ward.				
Psychological tensions	15	Working in the COVID-19 ward has made me angry	**(4)**	**(3)**	**(2)**	**(1)**
	16	The inappropriate behavior of people around me makes me angry and upset				
	17	My children stay away from me because of the fear of contracting COVID-19.				
	18	I am embarrassed to work in the COVID-19 ward.				
	19	I am afraid of infecting the people around me with the COVID-19 virus.				
	20	Working in the COVID-19 department has made me feel lonely and isolated.				

**Table 9:** The score range of each class of the measurement scale for the Social stigma of COVID-19 in nurses

Dimensions	Min. score	Max. score	Unfavorable	Moderate	Favorable
social well-being	7	28	7-13.9	21 − 14	28-21.1
Rejection	7	28	7-13.9	21 − 14	28-21.1
Psychological tensions	6	24	6-11.9	12-17.9	24 − 18
The whole scale	20	80	20-39.9	60 − 40	80-60.1

## Discussion

The present study aimed to design and psychometrically evaluate the COVID-19 social stigma questionnaire in nurses. The final questionnaire was extracted as 20 items and three dimensions, including social well-being, rejection, and psychological tension. The evidence showed acceptable validity and reliability for the questionnaire. The first dimension of the questionnaire was social well-being. The nurses stated that they valued themselves despite social stigma and were proud of themselves for working as nurses at COVID-19 wards. Consistent with our results, Qurbani and Shali claimed that appreciation and encouragement were people’s reactions in dealing with the treatment group during the pandemic [29]. Social support is an effective facilitator for psychological well-being in stressful situations. In addition, understanding the social and psychological support received from the family leads to a deep sense of value and gratitude [30]. Mostafa et al. (2021) adapted the SARS stigma scale to psychometrically evaluate a 16-item scale for the COVID-19 stigma among Egyptian doctors. This tool comprised three factors, namely personal stigma (8 items), disclosure and public attitude concerns (5 items), and negative experiences (3 items) [21]. In the dimension of disclosure and public attitude concerns in this tool, healthcare workers considered it wrong to tell others about their jobs and expressed regret. In contrast, in the questionnaire of the present study, it is stated in some items of the social well-being dimension: “Working in the COVID-19 ward made me known as a brave person in the eyes of companions”, “Patients appreciate us as nurses in the COVID ward”, and “the good attitude of colleagues motivates me to work”. This difference could be because the two studies were conducted in two different societies with various cultures. Besides, the current study focused on the attitudes of patients, family, friends, and colleagues. This difference may be explained by a highlighted emphasis on family values, cultural characteristics of the Asian region, and a strong sense of responsibility unique to healthcare workers [31]. Moreover, some of the items in this dimension of the present questionnaire are similar to those of Mostafa et al. (2021), e.g., “some people avoid me when they know that I am a health care worker” or “people are afraid of me because I am a healthcare worker” [21]. In Kasiani-Miranda’s questionnaire, some items are: “When I see news and stories about COVID-19 on TV, press, or social media, I get nervous or anxious” [32, 33]. Given the current coverage of social networks, mass media, and instant global communication via the Internet, the stigmatization phenomena promoted by these networks during the COVID-19 pandemic can be more considerable, even in populations with academic health education. The assessment of stigma levels between the general population and healthcare personnel indicated very similar percentages of stigmatization expressed by both populations regarding this aspect. In this respect, 43.4% and 42.9% stigma levels were determined in the general population and healthcare workers, respectively. Therefore, “infodemic” becomes a necessary and relevant factor. The phenomenon of COVID-19-associated stigma needs to be studied in more detail to determine its effects on different populations, including those of healthcare workers [34]. Moreover, our findings showed that mass media played an effective role in raising people’s level of knowledge and good attitudes toward nurses. This difference may result from different methods employed in studies in different research environments.

Rejection was the second dimension of the current questionnaire. From the nurses’ viewpoints, the fear of infection and being a carrier caused other people (family, friends, neighbors, and colleagues working in other wards) to avoid and stigmatize them as COVID-19 carriers. Theoretically, the feeling of fear and the perceived risks of the pandemic are directly associated with the transmission speed and the death rate [35]. People with high fear or perceived risk of the COVID-19 pandemic can react irrationally and create and prolong the stigma associated with COVID-19 infection [20]. The Kasiani-Miranda instrument, which measures COVID-19-related stigma and fear, refers to the social isolation of people working in health services who are in contact with COVID-19 patients. The mentioned instrument also contains an item titled “I am afraid of being infected by health personnel I meet in public transport, on the street, or at home”. This result suggests a correlation between a high level of fear of disease and stigmatizing attitudes toward health workers. These items correspond to some items in the present study, indicating the avoidance of nurses’ families and friends.

The third dimension of the questionnaire denoted the nurses’ mental tensions. The items in this dimension of the questionnaire indicate that nurses experience tensions as feelings of discomfort, depression, fear, anger, irritation, loneliness, and humiliation due to the COVID-19-related stigma and distancing from other close people. These findings are consistent with those of studies conducted based on people’s real experiences in different countries. They found that COVID-19-related stigma was associated with factors such as fear caused by infection or quarantine, supernatural or religious beliefs, and self-shame or self-blame for contracting the disease.

Healthcare workers experienced the stigma associated with caring for COVID-19 patients. They were victims of the experiences of discrimination, such as forbidding them to enter their homes, verbal abuse, rumors against them, and social worthlessness [36]. According to the results of the Kasiani-Miranda questionnaire, people consider contracting COVID-19 shameful and a divine punishment [31]. Previous studies reported negative impacts of the fear associated with stigma and discrimination on the general health of patients with chronic diseases, such as mental illnesses, AIDS, tuberculosis, leprosy, and epilepsy [37–39]. The stigmatization of health workers is associated with their psychophysical health. The COVID-19-related stigma, experienced at high levels by healthcare workers, causes fear, anxiety, negative attitudes, ignoring behavior and rejection, and psychological discomfort, which can negatively affect their performance [25]. In a tool designed by Tsukuda et al. (2022), healthcare workers expressed their worries and anxiety about disclosing their workplace. They felt guilty and filthy because of their close contact with patients [40]. These items correspond to some items of the present study in which nurses were afraid of infecting others with the COVID-19 virus. In the present study, however, the nurses did not state that they felt filthy and guilty after contacting the patients; this difference may result from the different populations.

The prevention of stigma depends on controlling or treating coronavirus, increasing knowledge about the disease, countering the tendencies of those stigmatizing others, and supporting stigmatized people through emotional support and social policies [41]. This intricate task warrants an interdisciplinary and multi-level approach that can be well achieved through working in scientific networks [42]. The data collected during the assessment of stigmatization situations are the first input to direct the development of informed intervention strategies. These data describe the magnitude of the phenomenon and related variables in each specific cultural context [42].

A limitation of the current research is that it was conducted in Iran, and the data were collected in cultural contexts appropriate to this country. Therefore, the results cannot be generalized to other societies, and the tool should be evaluated and validated in other countries.

## Conclusion

Social stigma should be controlled in the health system, considering its negative influence on the employees’ health and caregiving quality. Healthcare workers have a special sense of responsibility that is different from that of ordinary people. Therefore, the perceived stigma may not be expressed externally but may be internalized, leading to a psychological burden. Fear, anxiety, solitude, isolation, and feelings of rejection can cause fatigue, job burnout, and decreased satisfaction and mental health in nurses.

The instrument of the present study is a valid and reliable scale for the quantitative assessment of COVID-19 social stigma in nurses. Also, it is the first tool with a sequential exploratory approach based on qualitative data of nurses’ experiences at COVID-19 wards in Iran. This scale can help managers and health policymakers implement necessary protective measures by evaluating the social stigma of COVID-19 in nurses and emerging infectious diseases possibly occurring in the future. Stress management education courses, resilience training, and psychological/psychiatric consultations will help nurses achieve adaptation using effective coping strategies in the shortest possible time. Overall, this study will be an important step to improve their levels of psychophysical health, quality of life, and patient care quality.

### Electronic supplementary material

Below is the link to the electronic supplementary material.

Supplementary Material 1

## Data Availability

The datasets generated and/or analyzed during the current study are not publicly available because individual privacy could be compromised. However, they are available from the corresponding author upon reasonable request.

## Abbreviations

COVID-19: Coronavirus-19
WHO: World Health Organization
EFA: Exploratory factor analysis
CVR: Content validity ratio
CVI: Content validity index
KMO: Kaiser-Meyer-Olkin
PAF: Principle-axis Factoring
ICC: Intraclass Correlation Coefficient
PCA: principal component analysis
